# Hierarchical Structure and Thermal Property of Starch-Based Nanocomposites with Different Amylose/Amylopectin Ratio

**DOI:** 10.3390/polym11020342

**Published:** 2019-02-15

**Authors:** Shuyan Zhang, Jie Zhu, Yujia Liu, Shui-Yang Zou, Lin Li

**Affiliations:** 1 School of Chemical Engineering and Energy Technology, Dongguan University of Technology, Dongguan 523808, China; zhangsy@dgut.edu.cn (S.Z.); yujialiu@dgut.edu.cn (Y.L.); zousy@dgut.edu.cn (S.-Y.Z.); 2 Ministry of Education Engineering Research Center of Starch & Protein Processing, Guangdong Province Key Laboratory for Green Processing of Natural Products and Product Safety, School of Food Science and Engineering, South China University of Technology, Guangzhou 510640, China

**Keywords:** starch-based nanocomposites, amylose/amylopectin ratio, hierarchical structure, thermal property

## Abstract

Starch-based materials with reinforced properties were considered as one of the most promising materials to replace the petro-based packaging products, and actually, the molecular structures of starch usually determined the structures and properties of end-used starchy products. Here, starch-based nanocomposites were fabricated by starch esters derived from native starches with different amylose contents and organically modified montmorillonite (OMMT). The fractured surface under scanning electron microscopy (SEM) exhibited wrinkles formed by macromolecular aggregation owing to the interaction competition between the plasticizer and nanofiller with the starch ester. The more intense interaction within amylopectin-rich films promoted the formation of much randomly exfoliation of OMMT observed by Transmission electron microscopy (TEM). As the amylose content increased, the interaction between the starch ester and the nanofiller was weakened, leading to the dispersion morphology of an ordered arrangement and partly intercalated structures in the dimension of 12.92 to 19.77 nm. Meanwhile, such interaction also affected both the inner ordered structure integrity of starch ester and the layer structure consistency of nanofiller according to X-ray diffraction results. Further, the stronger interaction between amylopectin and the nanofiller endowed higher thermal stability to the amylopectin-rich starch-based nanocomposites. In short, these results are beneficial for the application of starch-based nanocomposites in the food packaging industry by regulating the interaction between starch and nanofillers.

## 1. Introduction

The demand for bioplastics in packaging is expected to rise at an estimated compound annual growth rate of 33% in the coming decades, as these materials are becoming cheap with improvements in their packaging properties [1]. Therefore, bio-based materials derived from starch, cellulose, gluten and chitosan are rapidly being used by the food packaging industry owing to their low cost and availability from multiple sources [2,3,4]. Moreover, to improve the mechanical and barrier properties of these materials resulting from the hydrophilicity of native starch [5], chemical modifications such as esterification, oxidation and etherification have been performed to decrease the drawbacks of starchy products [6,7,8,9]. In particular, esterified starch with different degrees of substitution (DSs) could effectively improve mechanical properties and hydrophobicity in humid environments [10,11,12].

In addition, the distribution of additive fillers with a high aspect ratio on the nanoscale can substantially lead to the enhancement of mechanical, rheological, gas barrier and optical properties of corresponding nanocomposites [13,14,15]. Such features make nanocomposites as ideal candidates for a broad range of application in high-barrier packaging materials [16]. Nanofillers such as clays (natural and quaternary ammonium-modified montmorillonite, sepiolite and halloysite) [17,18,19,20], metal ions (silver, copper, gold) [21], metal oxides (TiO_2_, ZnO, MgO) [22], starch nanocrystals [23] and cellulose nanocrystals [24,25] have been widely applied in starch-based materials. It has been indicated that the incorporation of starch/plasticizer/clay could generate a large matrix/filler interfacial area within the starch-based film, promoted a uniform dispersion of nanofillers into the polymer matrix, which substantially impacted the overall properties of the resulting nanocomposites [26,27,28].

Starch consists of two polysaccharides: linear amylose and highly multiple-branched amylopectin [29]. Studies have shown that the structures and properties of starch-based materials are determined by the molecular structures of starch and/or modified starch within the matrix [14]. For instance, many crystallites were formed in modified starch and starch-based film with a high content of amylose, which displayed high stiffness and breaking strength [30,31,32]. Therefore, the molecular structures of starch obviously influence the structures of end-use starchy products. Moreover, when nanofillers were added to plasticized starch-based films, the nanoparticles would interact with macromolecules to form different structural features, such as intercalated/exfoliated/phase-separated structures generated from the interaction between nanoparticles and amylose [33,34]. Concerning the potential application of starch-based nanocomposites in the food packaging materials, it is important to probe the effect of the interaction between nanofiller and starch, as well as its effect on the hierarchical structure of starch-based nanocomposites. Therefore, a discrepancy of the interactions between amylose/amylopectin and nanofiller are indispensable, which would further affect the structures and thermal properties of starch-based films.

Our previous studies characterized the multilevel structures of propionylated starches with different amylose/amylopectin ratios [30,31]. In this work, starch-based nanocomposites were prepared with starches containing various amylose contents. The detailed structural differences in the resultant films derived from the nanofiller/starch/plasticizer interaction were discussed to reveal how amylose or/and amylopectin determined the hierarchical structures of starch-based nanocomposites, which will help the development of starch-based materials for food packaging applications.

## 2. Materials and Methods 

### 2.1. Materials 

Low-amylose maize starch (LA) and medium-amylose maize starch (MA) were obtained from Lihua Starch Co., Ltd. (Qinghuangdao, China) and Huanglong Food Industry Co., Ltd. (Jilin, China), respectively. High-amylose maize starch (HA) was supplied by Penford Australia Pty Ltd. (Lane Cove, NSW, Australia). Propionic anhydride and pyridine were prepared from Sinopharm Chemical Reagent Co., Ltd. (Shanghai, China). Triacetin and acetone purchased from Aladdin Chemistry Co. Ltd. (Shanghai, China). Dellite 72T (OMMT), a montmorillonite organically-modified by dimethyl-dihydrogenated-tallow ammonium, was supplied by Laviosa Chimica Mineraria S.P.A (Livorno, Italy), the particle dimension was 15–20 μm by laser particle analyzer (Coulter LS13320, Beckman Coulter, Inc., Beckman, CA, USA). All chemicals and solvents were used in analytical grade.

### 2.2. Preparation of Different Amylose Content Starch-Based Nanocomposites

Three species of propionylated starch esters with high degree of substitution (DS) were prepared with the same method as described in the previous study [30], i.e., LA (low amylose content) starch ester (DS = 2.61 ± 0.03), MA (medium amylose content) starch ester (DS = 2.35 ± 0.01), and HA (high amylose content) starch ester (DS = 2.50 ± 0.03). Triacetin (*Mw* = 218.20) and acetone were selected as the plasticizer and solvent, respectively.

Starch-based nanocomposites were prepared using a solvent-casting method referred to in our previous study [11,31]. In the beginning, a measured amount of OMMT (4%, *w*/*w*, dry base) was dispersed in acetone by vigorous stirring. Then propionylated starch ester (1 g) was added and mixed with stirring. According to our previous results [11], 30 wt % triacetin (*w*/*w*, mass of starch) was added. The resultant solution was cast into a polypropylene plate, and resulting products were obtained for drying overnight in a vacuum oven at 45 °C for 12 h, the thickness of starch-based nanocomposites was measured by micrometer caliper: 110 ± 5.4 μm (LA), 123 ± 10.2 μm (MA) and 131 ± 8.6 μm (HA).

### 2.3. Characterization

#### 2.3.1. Morphology 

The cryo-fractured surfaces of nanocomposites were used for SEM analysis by an EVO 18 scanning electron microscope (Carl Zeiss Microscopy, GmbH, Oberkochen, Germany). The operating voltage was 20.0 kV. To avoid charging during scanning, the specimens were sputter-coated with gold prior to the experiment, and the samples were observed at a magnification of 1000×.

#### 2.3.2. Transmission Electron Microscopy Analysis

The films were cut into strips (1 mm × 2 mm), and then, they were embedded in a paraffin block and ultramicrotomed under cryogenic conditions (−50 °C) with a thickness of ~100 nm. The selected sections were deposited on a 300 mesh copper grid with a carbon coating. The distribution of OMMT within the film matrix was observed in a bright field imaging mode by JEM-2100F transmission electron microscope (JEOL, Tokyo, Japan). The acceleration voltage was 220 kV, and the magnification was 50,000×.

#### 2.3.3. Small-Angle X-Ray Scattering (SAXS) Analysis 

The nanocomposites were cut into strips for SAXS experiment using a SAXSess camera (Anton-Paar, Graz, Austria). A PW3830 X-ray generator with a long fine focus sealed glass X-ray tube (PANalytical) was utilized, and the operating voltage and current were 40 kV and 50 mA, respectively. A block collimator provided an intense monochromatic primary beam (Cu-Kα, *λ* = 0.1542 nm). The films were placed in the sample holder along the line shape X-ray beam in the evacuated camera housing. The sample-to-detector distance was 261.2 mm, and the temperature was kept at 26.0 °C. The 2D data were integrated into the one-dimensional scattering function *I*(*q*), as a function of the magnitude of the scattering vector *q* defined as Equation (1):

*q* = 4πsin*θ*/*λ* (1)

*λ* is the wavelength and 2*θ* is the scattering angle. Each measurement was collected for 5 min. All *I*(*q*) data were normalized to have the uniform primary intensity at *q* = 0 for transmission calibration. Desmearing was necessary because of the line collimation. 

#### 2.3.4. Wide angle X-ray Diffraction (XRD) analysis 

To study the crystallography of the nanocomposites, X-ray diffraction patterns (X’Pert PRO, PANAlytical, Almelo, Holland) were used with Cu-Kα radiation (*λ* = 0.1542 nm). The voltage was 40 kV and the current was 40 mA. Scattered radiations were selected and parallelized in the range of 2*θ* = 5–40°, at a step size of 0.033° at room temperature, counting 15 s. The basal spacing (*d*) of the nanofiller was calculated using the Bragg’s law (nλ = 2*d*sinθ), where θ is the diffraction angle and *λ* is the wavelength, and *n* is an integer.

#### 2.3.5. Thermal Property

Thermal analysis of nanocomposites was carried out using thermogravimetric analysis (TGA) (Perkin Elmer, Waltham, MA, USA). Samples were heated from 25 to 500 °C at a heating rate of 10 °C/min. Nitrogen was used as the purge gas at a flow rate of 25 mL/min.

#### 2.3.6. Statistical Analysis

All data were presented as the mean ± standard deviation (S.D.). Differences between groups were estimated by analysis of One-way ANOVA test using the SPSS software (version 19.0, IBM Co., New York, USA), and *p* < 0.05 was considered to indicate a statistically significant difference.

## 3. Results and Discussion

### 3.1. Morphological Characterization

**Figure 1:**
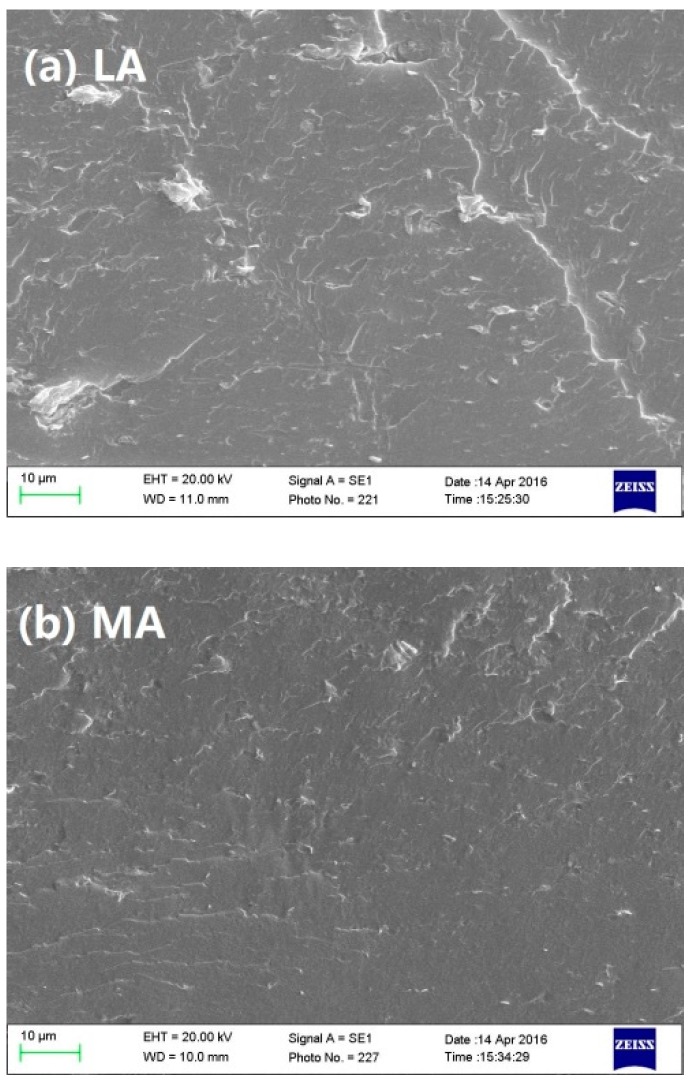

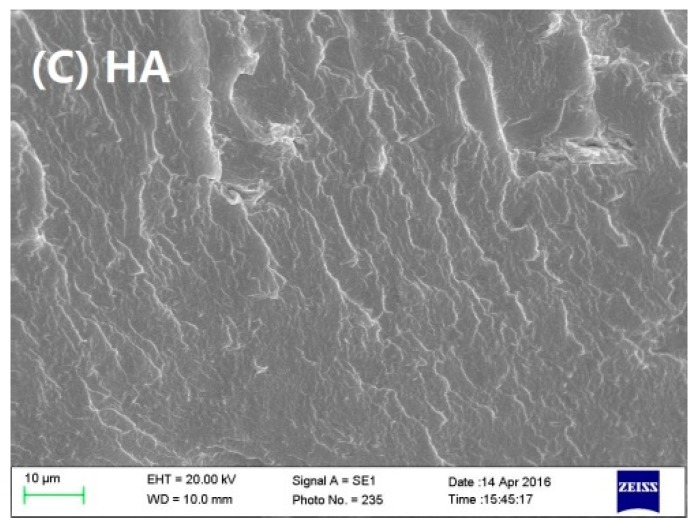
Morphology of the fractured surfaces of starch-based nanocomposites with (**a**) low-amylose content (LA), (**b**) middle-amylose content (MA) and (**c**) high-amylose content (HA).

The cryo-fractured surfaces of the starch-based nanocomposites with different amylose contents were characterized by SEM (Figure 1). It was previously reported that propionylated starch with high *DSs* (2.27 and 2.61) tended to form smooth surfaces [35], whereas others have disclosed that, with a high content of amylose, starch-based films displayed an increased surface roughness [36]. In the present work, generally, the continuous cross-sections were displayed and implied a well interfacial adhesion between nanofiller and starch matrix. However, white granules and small aggregates were scattered throughout the matrix of starch-based films; some rough textures were still observed in the HA film. To comparatively speculate, the linear amylose could more easily interact with each other to facilitate the formation of compact structures. This competitive behavior necessarily weakened the interaction between the nanofiller and starch, which could lead to more heterogeneously wrinkled structures.

### 3.2. Dispersion of the Nanofiller

**Figure 2:**
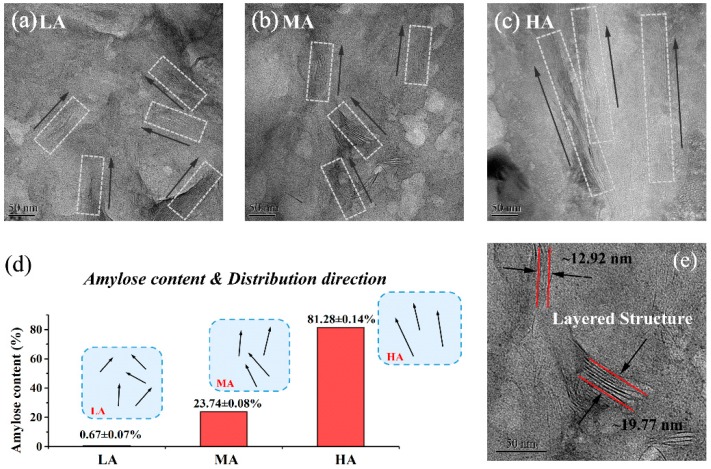
Nanofiller dispersion in the starch-based nanocomposites with (**a**) LA, (**b**) MA, and (**c**) HA; (**d**) the diagram of amylose content and dispersion direction; (**e**) layered structures of nanofiller.

TEM is one of the best techniques for exploring the morphology of clay contained nanocomposites. It is used to observe the dispersion of nanolayers at macroscale and microscale intuitively. Figure 2 showed the distributions of OMMT in starch-based films at 50,000 magnifications. In Figure 2a,b, TEM pictures showed almost individually dispersed particles, and nanolayers had disordered distributions in the matrix of LA and MA films. In addition, some layered structures at the scales of ~13 nm and ~20 nm were observed in MA film as labeled in Figure 2e. Comparatively, as shown in Figure 2c, stratified structures were showed in HA film. In this case, it seemed like the OMMT dispersed in the light of single-directional arrangement rather than aggregation on large scale.

Furthermore, the relation between observed distributions of nanofiller and amylose contents of the films was shown in Figure 2d. Previous research has observed the morphology of well-dispersed nanofiller throughout the polymer matrix [36], and there were heterogeneous dispersion regions. Other researchers have reported that high plasticizer content would induce some phase separation [35,37,38], which would correspond to our results of starch-based nanocomposites with 30 wt % triacetin. These comparisons revealed that the distribution of nanofiller within the starch matrix gradually changed from multi-directional to single-directional with the amylose/amylopectin ratio increased. Our previous works have shown that starch esters with high amylose content displayed more ordered regions than low-content amylose as well [30,33]. Conversely, highly-branched starch ester with side chains would more easily participate in the formation of entangled structures, which could cause strong interaction with nanofiller and facilitated the destruction of layered structures. Therefore, the multi-directional distribution and exfoliated structures would be generated. Moreover, as the amylose content increasing, more-ordered regions in HA film matrix probably weakened the interaction between the linear amylose and OMMT, therefore, single-directional distribution was performed and layered structures were retained, which probably resulted in more intercalation and even phase-separation.

### 3.3. Microregion Structures

**Figure 3:**
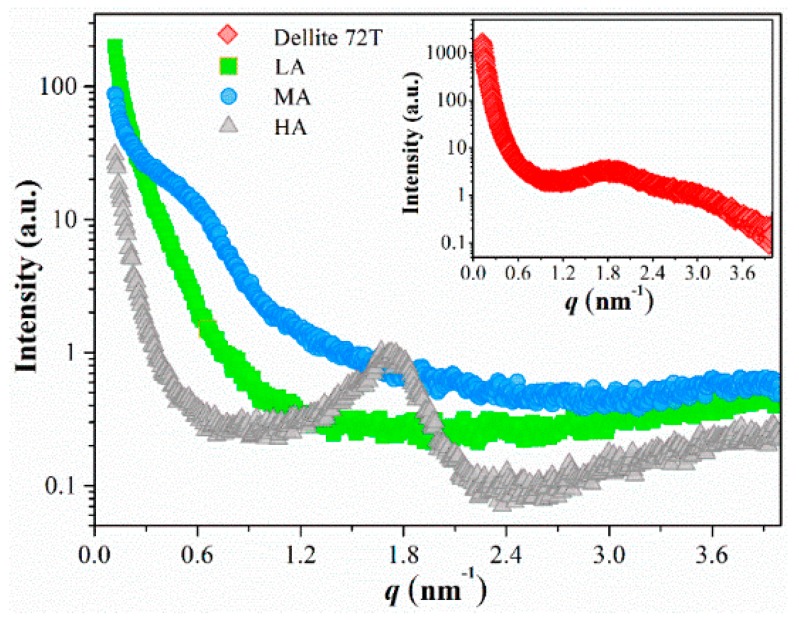
SAXS plots of OMMT and starch-based nanocomposites with different amylose/amylopectin ratios.

Microregion aggregation structure was organized by the arrangement of ordered and amorphous structures in the starch-based film at a large nanometer scale [35]. Figure 3 shows the discrepancy in the scattering intensity among starch-based nanocomposites with different amylose/amylopectin ratios. 

Thereinto, the SAXS spectrogram of OMMT was shown in the inset, which displayed an obvious peak at *q* = 1.81 nm^−1^, this peak was corresponding to a *d*-spacing scale of 3.47 nm calculated by the Bragg’s law. A Previous study has shown that amylose to amylopectin ratio rather than the sources of starch (corn, potato, wheat) affected the *d*-spacing [39]. In the present work, when OMMT particles were incorporated in LA and MA films, the characteristic peak of nanofiller both disappeared. It was speculated that the main peak loss of clay on a large scale was attributed to the disruption of highly-branched starch. Others also have reported that the starch ester chains with highly-branched density could accelerate the exfoliation of clay platelets and low concentration of clay agglomerates [18,40,41]. Therefore, the interaction between highly-branched starch esters and OMMT could promote the destruction of interlayer structures, resulted in a disordered distribution of nanofiller, as evidence from TEM in Figure 2. The exfoliated structures were mainly formed in LA film.

Meanwhile, a small shoulder peak was observed at approximately *q* = 0.50 nm^−1^ (*d* = 12.56 nm) in the MA film with the amylose/amylopectin ratio increased. This indicated some ordered structures were newly formed in this film, which was consistent with the layered structures marked in Figure 2e. For HA film, the characteristic diffraction peak was observed at approximately *q* = 1.70 nm^−1^ (*d* = 3.69 nm), which shifted toward the smaller *q* region than that of OMMT. These increasing *d* values indicated that the starch ester chains interpenetrated into the adjacent layers of OMMT, and enlarged the basal spacing of clay, which signified a greater degree of intercalated structure in HA film. In conclusion, it could be deduced that highly-branched starch esters presented more conspicuous noncovalent interactions with the nanofiller than linear starch (amylose), which would promote the formation of exfoliated structures.

### 3.4. Crystalline Structure

**Figure 4:**
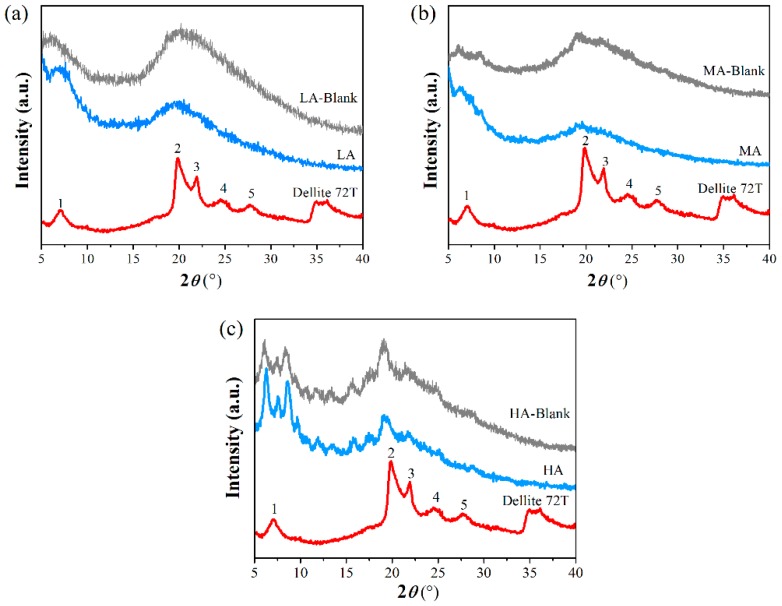
XRD data of starch-based films and OMMT (the curves have been shifted vertically to avoid overlapping). (**a**) LA, (**b**) MA, and (**c**) HA. “–Blank” represented for the corresponding starch-based films without nanofiller.

Figure 4 showed the XRD patterns of starch-based nanocomposites with different amylose/amylopectin ratios. The potential changes in the crystalline structures after the addition of OMMT were analyzed. Several major peaks for OMMT were observed at 2*θ* = 6.85° (*d* = 12.89 Å), 19.84° (*d* = 4.47 Å), 22.01° (*d* = 4.06 Å), 24.58° (*d* = 3.64 Å) and 27.59° (*d* = 3.62 Å). Comparatively, with OMMT incorporation in LA film, the characteristic peaks of OMMT all disappeared, and the widths of peaks simultaneously were broadened, which was also the same for MA film. As the amylose content increasing, the addition of OMMT did not alter the HA-blank film peak patterns as shown in Figure 4c, the diffraction peaks for OMMT were also generally maintained.

These changes in XRD pattern could be understood as following steps. Firstly, the platelets of OMMT lost the ordered crystalline structure and became disordered, which could promote a peak with broader and wider distribution [42]. Then, an increasing number of polymers penetrated into the space between the clay galleries, resulting in the shift of XRD peak toward a smaller diffraction region. Finally, the peak became increasingly wider, more clay platelets were pushed apart and exfoliated [41,43]. Our TEM results showed that the well-dispersed clay layers could be easily trapped into starch molecules with multi-branched structures. According to the kinematical scattering theory, the broader peaks were caused by either reduced crystal size or the presence of large defects [34], and the exfoliated nanocomposites could produce the greatest surface area interaction between clay nanoplatelets and fine polymers [44]. Thus, destruction of the crystal interlayer structure of OMMT was attributed to being promoted by the interactions with highly-branched starch esters and further led to the formation of exfoliated structure. Comparatively, within the nanocomposites with high amylose content, chains with a high degree of chain-chain interactions allowed the formation of entangled networks, which might not favor the dispersion and exfoliation of nanofiller. Nevertheless, only short outer chains of waxy starch participated in the formation of weak entangled structures [33], the interaction between OMMT and linear starch ester was inadequate. Thus, the layer structure of OMMT and the crystallites in HA film were retained.

### 3.5. Thermal Properties

**Figure 5:**
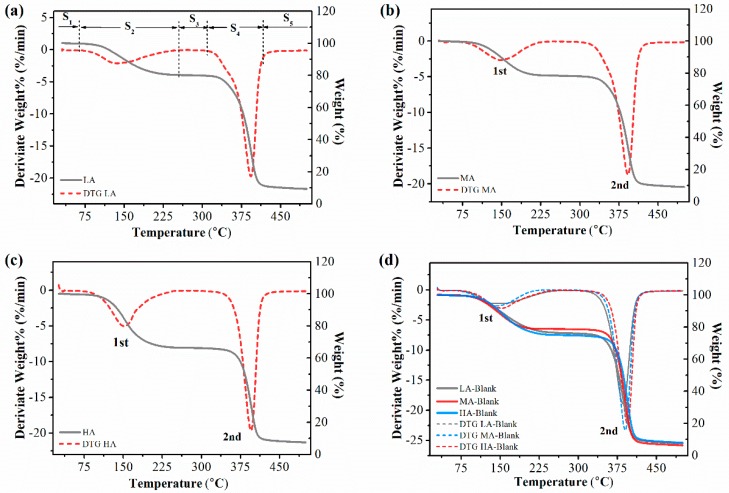
Thermogravimetric curves of starch-based nanocomposites with different amylose/amylopectin ratios and the control samples.

The thermal stabilities of starch-based nanocomposites were investigated by thermogravimetric analysis. The film degradation processing displayed five stages (Figure 5). The first stage was corresponding to the loss of adhesive water, which could be ignored in this study due to the hydrophobic property of the starch-based films. The second stage, at the range of 60–250 °C, was the vaporization of triacetin, which has been disclosed in our previous research [12]. There was no decomposition in the third stage (from 250 to 350 °C). The depolymerization and pyrolytic decomposition of the polysaccharide backbone were related to the fourth stage, at 350–450 °C and the last stage was similar to the third one, meaning that only ash was left [12,45].

The peak temperatures of starch degradation with and without OMMT addition were shown in Table 1, which showed improved thermal degradation temperature for nanocomposites with nanoclay addition. This higher temperature was reasonably attributed to the higher aspect ratio (width to thickness) of the nanofiller acting as a heat barrier, and meanwhile, a carbonaceous-silicate char built up on the film surface, which restricted polymer chain mobility through the nanoclay galleries [19,40,46].

On the other hand, as shown in Table 1, the degradation temperature increased as the amylose content increased for both starch-based films with and without OMMT. The previous works have found that the plasticized amylose could favor some crystallites formation and therefore restricted the macromolecular motion, which was presented as high thermal stability [30,31]. However, it was interesting that, compared to the films without OMMT, there was the greatest degree of increase for the degradation temperature of LA film (6.80 ± 0.73 °C), while only 1.21 ± 0.47 °C for HA film. This result could demonstrate that, compared with amylose starch, OMMT interacted with branched amylopectin and enhanced the thermal stability of the corresponding nanocomposites to a greater extent. As discussed in this work, the highly-branched amylopectin facilitated the dispersion of individual nanoparticles, and that would mean more heat barrier was uniformly dispersed within the film matrix. Therefore, this homogeneous phase would increase the improvement of thermal stability by restricting the macromolecular motion everywhere. By contrast, more crystallites in HA film could promote some intercalation structures or even phase separation, and the limited geometrical constraints alleviated the improvement of thermal stability.

## 4. Conclusions

Since starch-based nanocomposite is one of the most promising novel materials, the molecular structures of the starch macromolecule and the nanofiller should positively affect the structures and properties of the end-used products for different food application. 

In this work, we prepared starch-based nanocomposites with organically-modified montmorillonite and hydrophobic propionylated starches containing different amylose contents. The different interaction between nanofiller and amylose/amylopectin would determine the hierarchical structures (morphology, nanostructure, and crystallites) and thermal property of the films. Due to the branched molecular structure, amylopectin tended to facilitate nanoparticles with more favorable dispersion and destruction of layered structures of nanofiller, which would finally generate the exfoliation structure within the film matrix. Whereas the plasticized linear amylose showed more obvious crystallization, and thus more crystallites and compact structures would not afford sufficient interaction between amylose and nanofiller, which led to the intercalation structures and even some phase separation. In consequence, the LA film with homogenous nanofiller dispersion displayed a greater degree of improvement of thermal stability. Therefore, this work could not only benefit understanding the effect of raw material molecular interaction on the hierarchical structures and thermal property of resultant nanocomposites but also provide a feasible way of design novel starch-based materials in the food packaging industry.

## Figures and Tables

**Table 1:** Peak temperature values (°C) of starch-based composites with and without nanofiller.

Sample	Peak 2	Sample	Peak 2′	Δ*T*
LA-Blank	386.12 ± 0.81	LA	392.92 ± 1.04	6.80 ± 0.73 ^a^
MA-Blank	390.55 ± 1.25	MA	392.96 ± 1.16	2.40 ± 1.87 ^b^
HA-Blank	393.35 ± 0.95	HA	394.56 ± 1.35	1.21 ± 0.47 ^b^
